# 

**Published:** 1888-08

**Authors:** 


CONTENTS

VOL. 35 No. 8.

| | PAGE. |
|---|---|
| Extremes in Healing | 169 |
| The History of Mind Cure | 171 |
| Opinions Differ | 177 |
| Fiendish Enterprise | 179 |
| A Georgia Wonder | 180 |
| Advice as to a Headache | 181 |
| Invisible Intelligences | 182 |
| Our Foes in the Air | 183 |
| Why we should Bathe | 184 |
| John Odlin Page | 185 |
| Live your Religion | 187 |
| Mrs. Browning's Warning | 187 |
| Book Notices | 188 |
| Household | 189 |
| Miscellaneous | 191 |

INDEX TO ADVERTISEMENTS OF HALF A PAGE AND OVER.

| | PAGE. |
|---|---|
| Crystal Pepsin Tablets | I |
| Yankee Blade | I |
| Stevensville Mills | II |
| The Tea Cure | II |
| The American Oxygen Association | III |
| Hollow Suppositories | IV |
| Lactopeptine | V |
| Premiums of Books | VI |
| J. Albert Kimball, D. D. S. | VII |
| Bovinine | VIII |
| Volapük | IX |
| Rio Chemical Co. | X |
| Page & Co. | XI |

